# Connecting Health and Technology (CHAT): protocol of a randomized controlled trial to improve nutrition behaviours using mobile devices and tailored text messaging in young adults

**DOI:** 10.1186/1471-2458-12-477

**Published:** 2012-06-22

**Authors:** Deborah A Kerr, Christina M Pollard, Peter Howat, Edward J Delp, Mark Pickering, Katherine R Kerr, Satvinder S Dhaliwal, Iain S Pratt, Janine Wright, Carol J Boushey

**Affiliations:** 1 Curtin Health Innovation Research Institute and the School of Public Health, Curtin University, Perth, WA, Australia; 2 Department of Health, Perth, WA, Australia; 3 Centre for Behavioural Research in Cancer Control, Curtin University, Perth, WA, Australia; 4 School of Electrical and Computer Engineering, Purdue University, West Lafayette, IN, United States; 5 School of Engineering and Information Technology, The University of New South Wales at the Australian Defence Force Academy, Canberra, Australia; 6 Cancer Council Western Australia, Shenton Park, WA, Australia; 7 Epidemiology Program, University of Hawaii Cancer Center, Honolulu, HI, United States; 8 Department of Nutrition, Purdue University, West Lafayatte, IN, United States

**Keywords:** Nutrition, Diet, Text messaging, Mobile phone, Randomised controlled trial, Tailoring

## Abstract

**Background:**

Increasing intakes of fruits and vegetables intake, in tandem with reducing consumption of energy-dense and nutrient poor foods and beverages are dietary priorities to prevent chronic disease. Although most adults do not eat enough fruit and vegetables, teenagers and young adults tend to have the lowest intakes. Young adults typically consume a diet which is inconsistent with the dietary recommendations. Yet little is known about the best approaches to improve dietary intakes and behaviours among this group. This randomised controlled trial aims to evaluate the effectiveness of using a mobile device to assess dietary intake, provide tailored dietary feedback and text messages to motivate changes in fruit, vegetable and junk food consumption among young adults.

**Methods/design:**

The CHAT project will involve the development of the mobile device food record (MDFR), and evaluation of dietary feedback and implementation of a 6-month intervention in young adults aged 18 to 30 years. The participants will be randomly assigned to one of three groups (1) Intervention Group 1: MDFR + Text Messages + Dietary Feedback; (2) Intervention Group 2: MDFR + Dietary Feedback; (3) Control Group 3: MDFR, no feedback. All groups will undertake a 3-day dietary record using the MDFR but only the Intervention Groups 1 and 2 will receive tailored dietary feedback at baseline and at 6-months which will consist of assessment of serves of fruits, vegetables and junk food in comparison to dietary recommendations. Tailored nutrition text messages will be sent to Intervention Group 1 over the 6 months. Data will be collected at baseline and again at the 6-month completion.

**Discussion:**

This trial will test if applications running on mobile devices have potential to assess diet, provide tailored feedback and nutrition messages as an effective way of improving fruit and vegetable consumption and reducing energy-dense nutrient poor foods in young adults. The CHAT project will assess the impact of the intervention on behavioural intention to eat a more healthful diet. This innovative approach if successful may provide a means to deliver a low cost health promotion program that has the potential to reach large groups, particularly young adults.

**Trial registration:**

Australian and New Zealand Clinical Trials Registry ACTRN12612000250831

## Background

Studies have linked poor diet quality and lack of variety with increased incidence of chronic diseases such as cardiovascular disease, some cancers and diabetes. Increasing fruit and vegetable consumption and limiting energy intake from total fats and sugars are dietary priorities to prevent chronic disease [1]. Inadequate consumption of fruit and vegetables is a major risk factor contributing to 1.8% of the worldwide burden of disease, with Australian estimates at 2.7% [2]. In the US the majority of the population consumes a diet low in fruits, vegetables and whole grains but over-consumes solid fats, sugars and alcoholic beverages [3]. The Australian National Nutrition Survey found that only 8% of Australian adults met the minimum 400 grams of fruit and vegetables recommended by WHO [4]. Only 24-30% of adults met the vegetable target of five or more 75 gram servings per day, compared to 30-37% meeting the recommended two 150 gram servings of fruit per day [5]. Adolescents and young adults were the sub-population group with the lowest fruit intake, while for vegetables about half of all adults were eating less than the recommended serves [6]. Australian adults consume 36 percent of their energy intake from energy-dense nutrient-poor (EDNP) foods and young men typically consume a higher proportion of these foods [7]. Excessive consumption of alcoholic beverages and binge drinking is also common amongst young Australians [8]. Despite the evidence that in Australia young adults consume a diet inconsistent with the dietary recommendations, little is known about the best approaches to improve their dietary intakes and behaviours.

Studies show that adults have an understanding of the relative healthfulness of foods. In Australia, people are comfortable with the term ‘junk food’ when describing foods and drinks of limited nutritional value. ‘Junk food’ or EDNP foods have been defined as any food (including beverages) high in fat, sugar and/or salt with little nutritional value or discretionary/non-core foods such as fast foods, sweetened breakfast cereals, confectionery or fizzy drinks [9,10]. The Australian Guide to Healthy Eating defines non-core foods as ‘extras’ or ‘discretionary’ and specifies the types of foods based on a 600 kiloJoules equivalent [11]. Before adults can improve the quality of their diet they need to have a greater awareness of the recommended number of servings of fruit and vegetables, as well as what constitutes EDNP foods [12,13]. However, many adults are unaware of what they are eating and think their diet is healthy when it isn’t [14,15]. Assessment of food intake in relation to dietary recommendations is necessary so that they are better informed on what constitutes a healthy diet consistent with dietary guidelines.

Assessment of food intake is a difficult task as it requires individuals to verbally describe or orally report in detail the food or beverage consumed and the amount they have had. The traditional dietary record requires individuals to keep detailed written accounts for 3–7 days of all food or drink eaten, including the portion consumed; either estimated in household measures (e.g. cups and spoons) or weighed with food scales. Food records require literate and highly motivated individuals. As this limits those willing to participate, a high degree of bias can occur. Adolescents and young adults, who typically have unstructured eating habits and frequently snack, are the least likely to undertake food records [16]. Obtaining a true picture of what a person eats is challenging. Using a mobile device to capture food intake has great potential to improve accuracy and reduce participant burden [16]. Currently the TADA project has been developing a Mobile Phone Food Record to assess dietary intake [17,18]. The mobile phone application uses a camera to capture before and after images of food and beverages consumed. These images are automatically uploaded to a server for analysis using a Wi-Fi connection. When taking the image, participants need to include a fiducial marker, which acts as a reference of known dimension and markings. The images are automatically analysed for characteristic features in food, such as colour, texture, and intensity, which are then used to identify a food. Together the information from image analysis and volume estimation can be linked to a nutrient database to estimate energy and nutrients consumed. The TADA system is currently being trialled in free-living populations but the full application with automatic image analysis is not yet available for public distribution. The CHAT application described in this paper has been adapted for use on a low-cost mobile device (iPod touch) so that the food images recorded by the participant are automatically uploaded to a server and the assessment of the images is undertaken by trained analysts. The automatic uploading of data will alleviate some of the burden to both participants and researchers. This project will use a food group based approach to provide tailored feedback to participants on their food intake in comparison to dietary recommendations. The over-optimistic perception of current fruit and vegetable intake finds many people believing that they already meet these dietary guidelines and not seeing the relevance of messages to eat more [12]. Providing accurate feedback on their current intake is important. Dietary assessment tools can be used to provide personalised descriptive feedback which can stimulate self-reflection on their eating behaviours [19]. This can assist people in making decisions about the need ‘or not’ to increase or decrease consumption.

Research on promoting dietary change has identified several factors known to be important. These are self-assessment of intake, tailored feedback and goals based on the diet assessment and reinforcement of behaviours with on-going key messages. People want messages which are personally relevant to them. Tailoring or personalising nutrition education messages has been shown to be effective in promoting dietary change [20-22]. Message-tailoring uses characteristics unique to the person to provide a message intended for that person rather than a group with the intention of improving behavioural outcomes [23]. Targeting or market segmentation of messages can be used to direct messages at a group level to reach subpopulations with certain unique characteristics [19]. In developing interventions it’s important to identify which approaches will be used in order to tease out which strategies are effective [23].

Developing the content of nutrition messages to assist people to understand and accept the importance of healthy eating is complex. Nutrition messages should be constructed to raise awareness of key health messages, increase motivation to perform the health behaviour and be relevant to the individual [24,25]. Personal experience and specific eating contexts should be incorporated to increase salience, for example, ‘packable’ or ‘quick and easy’ food [26]. Messages encouraging both individual and social responsibility are effective at increasing consumption [27,28]. The framing of messages is also important. Gain-framed messages which promote the benefits of engaging in a particular behavior are generally thought to be more effective than loss-framed messages for interventions focused on prevention [29]. The ultimate goal is to deliver messages which are persuasive and will lead to behavior change [30]. The additional challenges of delivery messages to a mobile phone is getting the correct content, frequency and length of message and sending at a time when the participant will be most receptive to receiving it.

The CHAT project aims to address the gaps in the existing literature by combining a dietary assessment tool in combination with nutrition messages sent to a mobile phone to support behaviour change. To our knowledge, there are no reported interventions where a mobile device in combination with a mobile phone has been used to measure food intake, provide tailored dietary feedback and support dietary behaviours through tailored nutrition messages. A small number of studies have used mobile phones to deliver health text messages in diabetes [31,32], weight loss [33,34] and smoking cessation [35] and promoting physical activity [36]. Tufano [37] in a review of eHealth (web and mobile phones) interventions for obesity pointed out that for health promotion interventions to be successful in promoting behaviour change they must reach the public. Mobile phones have the potential for a cost-effective and rapid communication of tailored nutrition messages. However, it’s critical that messages are carefully developed and the intervention is based on theoretical constructs. The CHAT project will provide participants with tailored feedback on their dietary intake in combination with tailored nutrition messages segmented by age, gender and behavioural characteristics and suitable for delivery via a text message. The purpose of the CHAT project is to develop a mobile device food record to assess serves of fruits, vegetables and junk (energy-dense nutrient-poor) foods and provide tailored feedback delivered in a timely manner to promote behaviour change in young adults. Additional on-going tailored messages will be delivered to the participants’ mobile phone to assist in the adoption of healthy eating behaviours. This paper describes the protocol of a randomised controlled trial to evaluate the effectiveness of using a mobile device to collect food intake data, provide tailored dietary feedback and tailored nutrition messages about fruit, vegetable and junk food delivered via a mobile phone to promote dietary change among young adults aged 18 to 30 years.

## Methods/Design

### Study design

**Figure 1:**
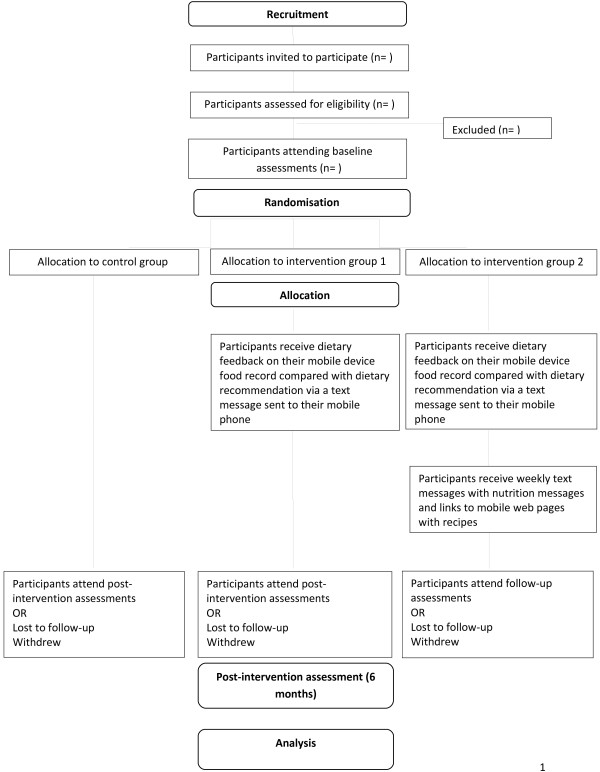
CHAT study design.

**Figure 2:**
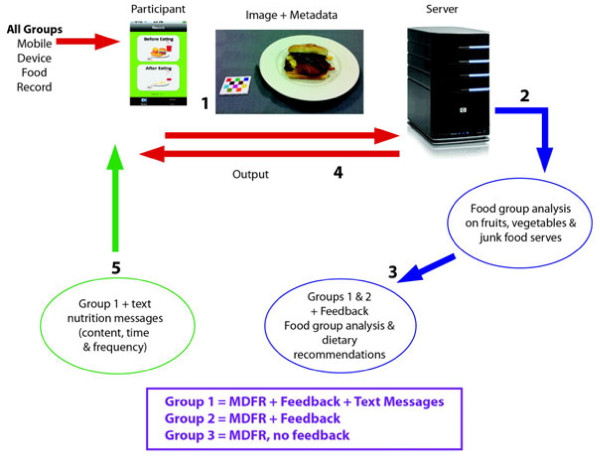
System architecture for the CHAT study Mobile Device Food Record (MDFR).

This project will consist of the development, implementation and evaluation of a 6-month nutrition intervention in young adults aged 18–30 years in metropolitan Perth, Western Australia. The study will be a 6-month randomised controlled trial (RCT) (Figure 1). The program has been designed to increase the intake of fruits and vegetables and decrease the intake of junk food through the use of dietary feedback and on-going motivational messages sent to the participant’s mobile phone. Figure 2 shows the system architecture for the study design. Data will be collected from participants at baseline and at the 6-month completion.

This intervention will use a mobile device to collect food intake data using an investigator-designed application running on an iPod touch. Each participant will be provided with a study iPod touch. The 3-day mobile device food record will be completed by all participants at baseline and at six months completion. Participants will take images of all food and drinks consumed over a continuous 3 day period. Intervention groups 1 and 2 will receive tailored dietary feedback on the number of serves of fruits, vegetables and junk foods consumed compared with recommended serves by a text message sent to their mobile phone. Intervention group 1 will also receive additional targeted nutrition messages via their mobile telephone (motivational messages on fruits, vegetables and extra foods or recipes) to promote dietary change. Feedback from focus group work indicates that participants recognise the term ‘junk food’ so this term will be used in communications with participants. The project protocol has been approved by the Curtin University Human Research Ethics Committee (approval number HR181/2011) and the Department of Health, Western Australia Human Research Ethics Committee (#2011/90).

#### Sample size

A sample size of 220 is required to estimate the proportion of subjects who will increase their fruit and vegetable intake to within a 95% confidence interval of +/− 7%. Allowing for a drop-out rate of 35%, 100 subjects will be recruited into each of the three treatment group. The sample size calculated for fruit and vegetable serves is based on the results of our pilot study and are conservative since the variability of the data (Standard Deviation) observed was about 4 times that reported in other studies.

### Recruitment

Participants from 57 suburbs (neighbourhoods) within in the Perth metropolitan area will be selected from the electoral roll in Western Australia. Suburbs will be selected to provide representation across socio-economic status (SES) based on the Socio-Economic Index for Area (SEIFA) [38] a value derived from income, education level, employment status and skill level. Using the Federal Electoral Roll, the first sample will contain 5000 names and addresses. A second sample will be used if required and will contain the same number or less, depending on the recruitment rate after the first mail out. The research team will not have access to the names and address data. These data will be delivered to a company approved by the Department of Health to conduct the mail out. After receiving a letter of invitation, participants who wish to take part in the study will contact the research team by email, mobile phone or the study website. Participants need to be aged between 18 to 30 years as of their last birthday and have a mobile phone. Participants will be excluded if (a) pregnant; (b) unable to complete the 6 month study; (c) undertake extreme forms of exercise or dieting; or (d) unable to attend the study centre on four occasions (two at baseline and two at completion).

Participants will undergo a telephone or on-line screening questionnaire. If they meet the selection criteria, once being informed of the details of the study and if willing to participate, they will be asked to attend a baseline data collection session at Curtin University which will include training on the use of the mobile device food record (MDFR). The study objectives and requirements will be explained to the participants who will complete an informed consent and have their height and weight recorded by the research staff. They will also complete a demographic and personal characteristics questionnaire. They will undertake the MDFR for three consecutive days (Thursday-Saturday). The following week, participants will return the mobile device and will then be randomized to one of three treatment groups. Sequence generation will be conducted by a biostatistician not involved in the implementation of the trial on site (therefore not in contact with the study participants). Research staff will not be involved in the randomisation or have access the randomisation sequence. Participants will be randomised to one of three treatment groups using a randomisation table created by computer software: (1) Intervention Group 1: MDFR + Text Messages + Dietary Feedback; (2) Intervention Group 2: MDFR + Dietary Feedback; (3) Control Group 3: MDFR, no feedback. Study participants or research staff cannot be blinded to the intervention. Research staff will need to liaise with the participants throughout the study.

Participants will complete additional questionnaires on their dietary habits and useability of the MDFR. Six months later they will be asked to repeat the 3-day MDFR and evaluation questionnaires. All three groups will record their food intake using the MDFR. The intervention groups will receive feedback on their food intake for serves of fruits, vegetables, junk food and alcohol. Participants will be forwarded dietary feedback and messages to their own mobile phones. Intervention Group 1 will receive additional weekly text messages on nutrition throughout the six months of the study. The control group will undertake the MDFR but will not receive feedback or text messages.

### Theoretical framework

The tailored intervention will be based on self-determination theory (SDT) and informed by motivational interviewing (MI) [39-41]. Recently Patrick and Williams suggested that combining SDT with MI may be useful in designing behaviour change interventions [41]. Central to SDT is the theory of human motivation which emphasises behaviours being autonomous (originating from one-self) as opposed to being controlled (pressured or coerced). The more intrinsic the motivation the more likely the person will engage in the behaviour because it’s something of importance or value to them [41]. As well as autonomy, competence (the need to feel capable of achieving the outcome) and relatedness (need to feel close and understood by others) are also considered psychological needs critical to internalisation. One of the principles of MI is that resistance will increase if the person perceives they are being coerced into a particular course of action [42]. This can occur when providing advice. When providing advice with SDT, it should be carried out in an autonomy-supportive manner with acceptance of the person’s decisions [40]. In relation to the intervention this emphasises the important of the tone used in all communications with participants. The tone and content of messages will be developed to support autonomous decision making. The intervention will also draw on the researchers past work which indicates the importance of providing practical solutions to barriers to healthy eating. These will include access to healthy, cheap, quick and easy to prepare recipes which have been adapted to a mobile phone platform.

### Message tailoring

Tailoring of nutrition messages is providing information that is relevant to the individual and therefore potentially more persuasive in changing eating behaviours [30]. Tailoring differs from targeting in that tailoring is based on characteristics specific to the person. The selection of messages will be tailored based on age group (18–22 or 23–30 years), gender, dietary intake and other behavioural and physical characteristics (BMI) known to influence eating behaviour. Tailoring of messages will occur for the outcomes being targeted: fruits, vegetables, junk food, alcohol and variety of fruits and vegetables. To illustrate how messages may be tailored, an individual who doesn’t consume alcohol will not receive messages on alcohol. If an individual is a young woman (18–22 years) who has indicated she is concerned about her weight, has a high consumption of junk food and a high BMI, then she will receive a set of messages relating to strategies around healthier options for snacks and meals. In keeping with the principles of motivational interviewing participants receiving nutrition messages will to able to opt out of specific content they do not wish to receive.

The delivery of the nutrition communication will make use of tailored nutrition messages at two levels. By the inclusion of two intervention groups the study will examine the effects of tailored dietary feedback based on the results from the participant’s dietary intake using the MDFR compared with the additional effects of tailored nutrition messages delivered to the participant’s mobile phone. As the act of recording food intake is known to influence dietary behaviour the control group will also complete the MDFR application but will not receive any feedback on their diet until after the intervention.

### Training on use of the mobile device food record

The MDFR has been developed by the research team at Purdue University [17,43]. The MDFR application was adapted for use in CHAT and allows for the automatic uploaded of food images collected by participants. When taking an image, participants need to include a scaling device known as a fiducial marker.

The training session on the use of the MDFR will take place at Curtin University in a room which has Wi-Fi access. All participants will be asked to keep a 3-day food record (1 weekend, 2 week days) with the special food recording application developed for a mobile device (iPod touch). Participants will undergo a briefing session on how to use the MDFR which will be used to record meals, drinks or snacks for a 3-day period.

The training session will be interactive and consist of a presentation using a 3D animated character which will include instruction on how to use the application (e.g. how to check if connected to Wi-Fi, how to capture food images). Previous research has shown that making the training session fun and interesting can improve cooperation and interest [17]. The animated character will be developed by a member of the project team (KK) who is multimedia designer and a 3D animator. The training will also include a practical session where participants will practice taking an image of a meal and successfully sending the image to the server. Participants will have a small booklet, designed to fit into the iPod carry case, for recording foods they forget to capture via the mobile device along with written and pictorial instructions on how to use the MDFR. All participants will receive a text message to remind them to start recording on the morning of the first day of their intervention and a second reminder in the evening to prompt them for any forgotten food. The following week, participants will return the mobile device and the research staff will verify the content of the images with participants (this is the normal procedure for paper-based food records). They will complete a questionnaire on the useability of the MDFR to guide the research team in future developments of the MDFR.

### Dietary feedback

The Intervention groups 1 and 2 will receive tailored feedback based on an assessment of their food intake from their MDFR. The participant’s 3-day diet will be assessed using a quality scoring of food items using food group analysis which will include serves and variety of fruits, vegetables and “junk food”, defined as foods and drinks high in fat, sugar and/or salt and alcohol with little nutritional value - or in colloquial food terms, fast foods, sweetened breakfast cereals, confectionery, fizzy drinks or alcohol [9,10]. Serving sizes for dietary assessment and feedback will be based on Australian Guide to Health Eating (AGHE) for fruits and vegetables (one serve of fruit is 150 g and one serve of vegetables is 75 g) and ‘discretionary food or extra serves (equivalents of approximately 600 kilojoules) [11]. Once the scoring is complete, the Intervention groups will be sent a text message with tailored feedback on the number of serves of food groups they consumed averaged over three days of recording, compared to recommended serves for adults from the AGHE. They will be prompted to set specific goals to increase serve of fruits and vegetables and reduce junk foods, where required.

### Nutrition messages to intervention group 1

#### Delivery

An automated text messaging delivery program, provided by a text messaging company [44] will be used to send text from the message library to the participant’s mobile phone during the six month intervention. Based on focus group work undertaken, four grouping variables have been selected: (1) young females aged 18–22 years; (2) older females aged 23–30 years; (3) young males aged 18–22 years; older males aged 23–30 years. The messages will be tailored to the grouping variable set up. At randomisation to this group, participants will be informed that they will be sent messages with content on fruits, vegetables, EDNP foods and drinks and alcohol. Participants will be given a choice to elect not to receive messages on particular content. This is based on the principles of motivational interviewing that unwelcome advice increases resistance [42]. Where multiple behaviours are involved, participants may be ready to change one behaviour but not another. For example, they may be willing to increase their fruit intake but unwilling to change their alcohol intake.

Participants are able to stop receiving text messages at any time by replying “stop”. If the participant send a message of STOP, the database will unsubscribe them immediately. There will also be additional categories to allow the participant to specify what messages they wish to stop receiving. For example if they send a message “no alcohol sms” they will no longer receive messages on alcohol.

In focus group work, participants requested healthy recipes. Past work has suggested that barriers to increasing fruit and vegetable intake has been the perceived cost and lack of cooking skills [45,46]. Recipes which were developed by the Department of Health in Western Australia have been adapted for easy readability and accessibility on a smart phone. Participants will receive text messages with links to recipes, which if they have a smartphone can be accessed from a mobile web page. For those without a smartphone they will be provided with a link to recipes on the study website. Pilot evaluation of the recipes suitability for delivery on a smart phone will be undertaken prior to the intervention.

#### Content

A library of approximately 50 messages is being developed. The content of the text messages is based on consumer knowledge, attitudes and beliefs that are known to influence food choices, and detailed message response testing in focus groups conducted in young adults aged 18 to 30 years, separated by age group (18–22 years and 23–30 years) and gender. In the first focus groups participants provided feedback on the tone and content of messages relating to fruit, vegetables, junk food and alcohol. Participants were asked to assess their preference for nutrition messages which would motivate them to eat healthily. For each of the areas of dietary behaviour change five different tonal messages were tested. Participants were asked to rank the messages for preference and were asked to identify the message most likely to persuade people to change how they eat.

Further focus groups in young adults were conducted to evaluate the final content, timing and dose of message delivery. This involved delivery the messages to participants on an iPhone and having them respond anonymously using polling software. A group discussion followed each message. A summary of the intervention content self-assessed by participants at baseline and at completion of the intervention is shown in Table 1.

**Table 1:** Summary of intervention content self-assessed by participants at baseline and at completion of the intervention

**Ate more or less**	**Substitutions**
**Ate more:**	**Switching to**
Servings of fruit and/or vegetables	Low energy/diet drinks or waters
Variety of fruits and vegetables	Lower-alcohol or non-alcoholic drinks
Adding more vegetables or salad to meals	Healthier options when eating out
	More alcohol free days every week
**Ate/drank less:**	
Sugary drinks (e.g. fizzy drinks, sports drinks or cordial)	
Confectionary (e.g. chocolate, lollies, cakes, sweet biscuits)	
Sugary foods (e.g. lollies, sugar in drinks)	
Fatty foods (e.g. pies, pastries)	
Alcohol	
Fast food	

#### Control group

The control group will record their dietary intake using the MDFR at baseline and again at 6 months completion. They will not receive any feedback on their food intake or any nutrition messages. As an incentive for retention in this group, they will be advised that they will receive feedback once the study has been completed.

### Outcome variables

The outcome variables measured at baseline and post intervention, will be the number of serves of fruits, vegetables and “junk food”. The data on the change in servings in each of the three groups will be compared using analysis of covariance. P-values < 0.05 will be considered as statistically significant. Possible covariates considered include age, gender and baseline value of the variable analysed. The differences between treatment groups in the mean change in intakes and 95% confidence intervals will be analysed and reported. Data will be analysed using IBM SPSS statistics version 19. The behavioural data will also be analysed using multiple logistic regression and generalised estimating equations (a technique used for repeated measures analysis of categorical data). At baseline and 6 months subjects will indicate their willingness to make changes to their fruit, vegetables, and or discretionary food intake and/or habits that assist these changes. This willingness to change will be assessed as ‘improve or not improve’ for fruits and vegetables and ‘reduce or not reduce’ for EDNP foods between the three treatment groups in the analyses. Odds-ratio and associated 95% confidence intervals will be reported. The analyses will also identify the characteristics of participants who are least likely to change their consumption of fruit and vegetables, and specified EDNP foods and drinks identifying target groups for future health promotion.

### Outcome measures

Participants will undertake all assessments at baseline and at the completion of the study at six months.

### Primary outcome measures

The serves of fruits, vegetables, junk and alcohol foods and beverages are the primary outcomes. Dietary intake will be assessed by the 3-day MDFR and scored for number of serves of fruits, vegetables and discretionary food according to the Australian Guide to Healthy Eating [11] standard serves as outlined previously by Rangan and colleagues [7].

### Secondary outcome measures and other covariates

Anthropometric variables of height and weight will be measured according to a standard protocol [47]. It is not expected that weight loss will occur as a result of the intervention.

Demographics and personal characteristics will be assessed by questions on gender, age, BMI, eating behaviour, educational level, country of birth, ethnicity, socioeconomic status, financial status, attitudes towards eating a healthy diet, perception of their body weight, intake of fruits, vegetables, junk and alcohol intake and recent dietary changes.

Eating behaviour will be assessed by *The 3-Factor Eating Questionnaire* which measures factors associated with eating behaviour: (a) 'cognitive restraint of eating', (b) 'disinhibition' and (c) 'hunger' [48].

Physical activity will be measured using *The International Physical Activity Questionnaire* (IPAQ) [49]. IPAQ has undergone extensive reliability and validity testing in 12 countries and has acceptable measurement properties for use in many settings.

Successful change in behaviour with regards to the primary outcomes will be examined by constructs of motivation, confidence, importance and intention to change for each of the key intervention target behaviours, fruits, vegetables, junk food and alcohol. Preference for autonomy will be assessed using a single item i.e., “In general, when it comes to my health I would rather an expert just tell what I should do” as developed by Resnicow et al. [50].

#### Process evaluation

Participants will be asked to provide feedback on the useability of the MDFR at baseline and post-intervention by a questionnaire adapted from *Useability Scale*[51]. Additional questions on the acceptability of the MDFR have been adapted from questionnaire tested in previous research [52]. In addition, participants will be asked to evaluate the dietary feedback (Groups 1 and 2) and the text messages (Group 1).

## Discussion

The CHAT project aims to determine if mobile devices can be used to deliver tailored feedback and nutrition messages as an effective way of improving fruit and vegetable consumption and reducing discretionary or non-core food intake in young adults. Adolescents and young adults in Australia are low consumers of fruit and vegetables with about half of all adults eating less than the recommended serves. Alcohol and EDNP foods are also an issue in this age group.

To promote good nutrition requires accurate understanding of the target group behaviour and the factors that influence it. Objective and accurate information on what is actually eaten is therefore vital. In addition understanding food intake and behaviour in the same context and terms as the consumer offers the building of behaviour change strategies.

The unique aspects of this study is that it will assess the impact of an intervention delivered via a mobile device on behavioural intention to eat more fruit and vegetables and reduce the intake of junk food in young adults aged 18 to 30 years. The tailored feedback provided to individuals may provide a cost effective strategy to deal with the barriers to improving eating behaviours in Australia, namely the misconception of adequacy of current intake through provision of dietary feedback data in comparison to current Australian recommendations. These proposed methods may build self-efficacy and promote behaviour change in fruit and vegetable intake and reduce junk food intake.

A novel aspect of this project is the use of tailored text messages on nutrition to support behaviour change. To our knowledge this is the first large trial of a text messaging intervention to promote healthy eating behaviours in young adults. The socio-demographic, physical characteristics and dietary behaviour data collected at baseline will be used to interpret the impact of receiving the text message on improving dietary behaviours. In future interventions it may be possible to tailor messages based on personal characteristics to make the messages more relevant to participants. This innovative approach if successful may provide a means to deliver a low cost health promotion program that has the potential to reach large groups, particularly young adults.

## Competing interests

The authors declare that they have no competing interests.

## Authors’ contributions

DK conceived and designed the study. DK and CP drafted the manuscript. All authors had input into the design of the study, intervention materials and the manuscript. SD contributed statistical expertise and generated the randomisation. KK designed the study logo and conceived and designed the recipe format. All authors read and approved the final manuscript.

## Pre-publication history

The pre-publication history for this paper can be accessed here:

http://www.biomedcentral.com/1471-2458/12/477/prepub
